# Supplementary material for: Regulation of S1P receptors and sphingosine kinases expression in acute pulmonary endothelial cell injury
Source: PeerJ. 2016 Dec 13;4:e2712. doi: 10.7717/peerj.2712 (PMC5157198; doi:10.7717/peerj.2712)
Supplement: Supplemental Information 1 [file peerj-04-2712-s001.docx]

**Table S1.** The change in TNF-α expression in HPAECs, at different time points using RT-PCR, in the presence of 1 μM LPS (**p* < 0.05).

|  | control | | | LPS | | |
| --- | --- | --- | --- | --- | --- | --- |
| 6h | 1.000 | 1.000 | 1.000 | 2.948 | 1.828 | 1.376 |
| 8h | 2.770 | 1.310 | 1.357 | 1.395 | 2.188 | 1.424 |
| 10h | 2.908 | 2.219 | 2.948 | 2.028 | 7.260 | 1.283 |
| 12h | 3.272 | 3.227 | 1.495 | 4.377 | 10.126 | 3.482* |
