# Supplementary material for: Regulation of S1P receptors and sphingosine kinases expression in acute pulmonary endothelial cell injury
Source: PeerJ. 2016 Dec 13;4:e2712. doi: 10.7717/peerj.2712 (PMC5157198; doi:10.7717/peerj.2712)
Supplement: Supplemental Information 2 [file peerj-04-2712-s002.docx]

**Table S2-1.** The change in expression of TNFα at different time points when injured HPAECs were co-cultured with MSCs using RT-PCR (*p<0.05, **p<0.01).

|  | control | | | MSC | | |
| --- | --- | --- | --- | --- | --- | --- |
| 8h | 1.000 | 1.000 | 1.000 | 0.921 | 0.695 | 0.732* |
| 16h | 1.000 | 1.000 | 1.000 | 1.505 | 1.214 | 1.200** |
| 24h | 1.000 | 1.000 | 1.000 | 1.169 | 1.257 | 1.084* |

**Table S2-2.** The change in expression of TNFα when injured HPAECs were co-cultured with different proportions of MSCs as examined at different time points by RT-PCR (*p<0.05, **p<0.01).

| Control | HPAEC:MSC 1:1** | HPAEC:MSC 1:2** | HPAEC:MSC 1:4* |
| --- | --- | --- | --- |
| 1.000  1.000  1,000 | 0.790  0.576  0.556 | 0.559  0.415  0.349 | 0.804  0.604  0.510 |
