# Supplementary material for: Regulation of S1P receptors and sphingosine kinases expression in acute pulmonary endothelial cell injury
Source: PeerJ. 2016 Dec 13;4:e2712. doi: 10.7717/peerj.2712 (PMC5157198; doi:10.7717/peerj.2712)
Supplement: Supplemental Information 3 [file peerj-04-2712-s003.docx]

**Table S3.** Change in expression of TNFα in injured HPAECs when treated with S1P and MSCs using RT-PCR (***p*<0.01).

| control | | | MSC** | | | MSC+S1P** | | |
| --- | --- | --- | --- | --- | --- | --- | --- | --- |
| 1.000 | 1.000 | 1.000 | 0.590 | 0.376 | 0.356 | 0.441 | 0.316 | 0.339 |
