# Supplementary material for: Regulation of S1P receptors and sphingosine kinases expression in acute pulmonary endothelial cell injury
Source: PeerJ. 2016 Dec 13;4:e2712. doi: 10.7717/peerj.2712 (PMC5157198; doi:10.7717/peerj.2712)
Supplement: Supplemental Information 4 [file peerj-04-2712-s004.docx]

**Table S4-1.** The expression change of S1P receptors 1, 2 and 3 following acute injury (**p*<0.05, ***p*<0.01).

|  | control | | | LPS | | |
| --- | --- | --- | --- | --- | --- | --- |
| S1PR1 | 1.000 | 1.000 | 1.000 | 1.515 | 2.928 | 2.222** |
| S1PR2 | 1.000 | 1.000 | 1.000 | 1.135 | 1.374 | 1.255 |
| S1PR3 | 1.000 | 1.000 | 1.000 | 1.257 | 1.885 | 1.571* |

**Table S4-2.** The expression change of sphingosine kinases 1 and 2 following acute injury (**p*<0.05, ***p*<0.01).

|  | control | | | LPS | | |
| --- | --- | --- | --- | --- | --- | --- |
| SphK1 | 1.000 | 1.000 | 1.000 | 1.266 | 1.905 | 1.586** |
| SphK2 | 1.000 | 1.000 | 1.000 | 1.905 | 1.945 | 1.925 |
