# Supplementary material for: Regulation of S1P receptors and sphingosine kinases expression in acute pulmonary endothelial cell injury
Source: PeerJ. 2016 Dec 13;4:e2712. doi: 10.7717/peerj.2712 (PMC5157198; doi:10.7717/peerj.2712)
Supplement: Supplemental Information 5 [file peerj-04-2712-s005.docx]

**Table S5-1.** Effect of MSC and S1P combination therapy on the expression change of TNF-α (** *p*<0.01).

| control | | | MSC** | | | 0.5μMS1P+MSC** | | |
| --- | --- | --- | --- | --- | --- | --- | --- | --- |
| 1.000 | 1.000 | 1.000 | 0.334 | 0.160 | 0.232 | 0.122 | 0.036 | 0.1530 |

**Table S5-2.** Effect of MSC and S1P combination therapy on the expression change of S1P receptors 1, 2 and 3 (** *p*<0.01).

|  | control | | | MSC | | | 0.5μMS1P+MSC | | |
| --- | --- | --- | --- | --- | --- | --- | --- | --- | --- |
| S1PR1 | 1.000 | 1.000 | 1.000 | 0.864 | 0.430 | 0.495** | 0.683 | 0.759 | 0.9740** |
| S1PR2 | 1.000 | 1.000 | 1.000 | 0.224 | 0.073 | 0.128** | 0.102 | 0.061 | 0.0625** |
| S1PR3 | 1.000 | 1.000 | 1.000 | 0.141 | 0.053 | 0.086** | 0.075 | 0.034 | 0.1760** |

**Table S5-3.** Effect of MSC and S1P combination therapy on the expression change of sphingosine kinases 1 and 2 (** *p*<0.01).

|  | control | | | MSC | | | 0.5μMS1P+MSC | | |
| --- | --- | --- | --- | --- | --- | --- | --- | --- | --- |
| SphK1 | 1.000 | 1.000 | 1.000 | 0.502 | 0.332 | 0.403** | 0.574 | 0.294 | 0.5510** |
| SphK2 | 1.000 | 1.000 | 1.000 | 1.033 | 1.049 | 1.035 | 0.534 | 0.593 | 0.7870** |
