# Supplementary figures and images for: Regulation of S1P receptors and sphingosine kinases expression in acute pulmonary endothelial cell injury
Source: PeerJ. 2016 Dec 13;4:e2712. doi: 10.7717/peerj.2712 (PMC5157198; doi:10.7717/peerj.2712)

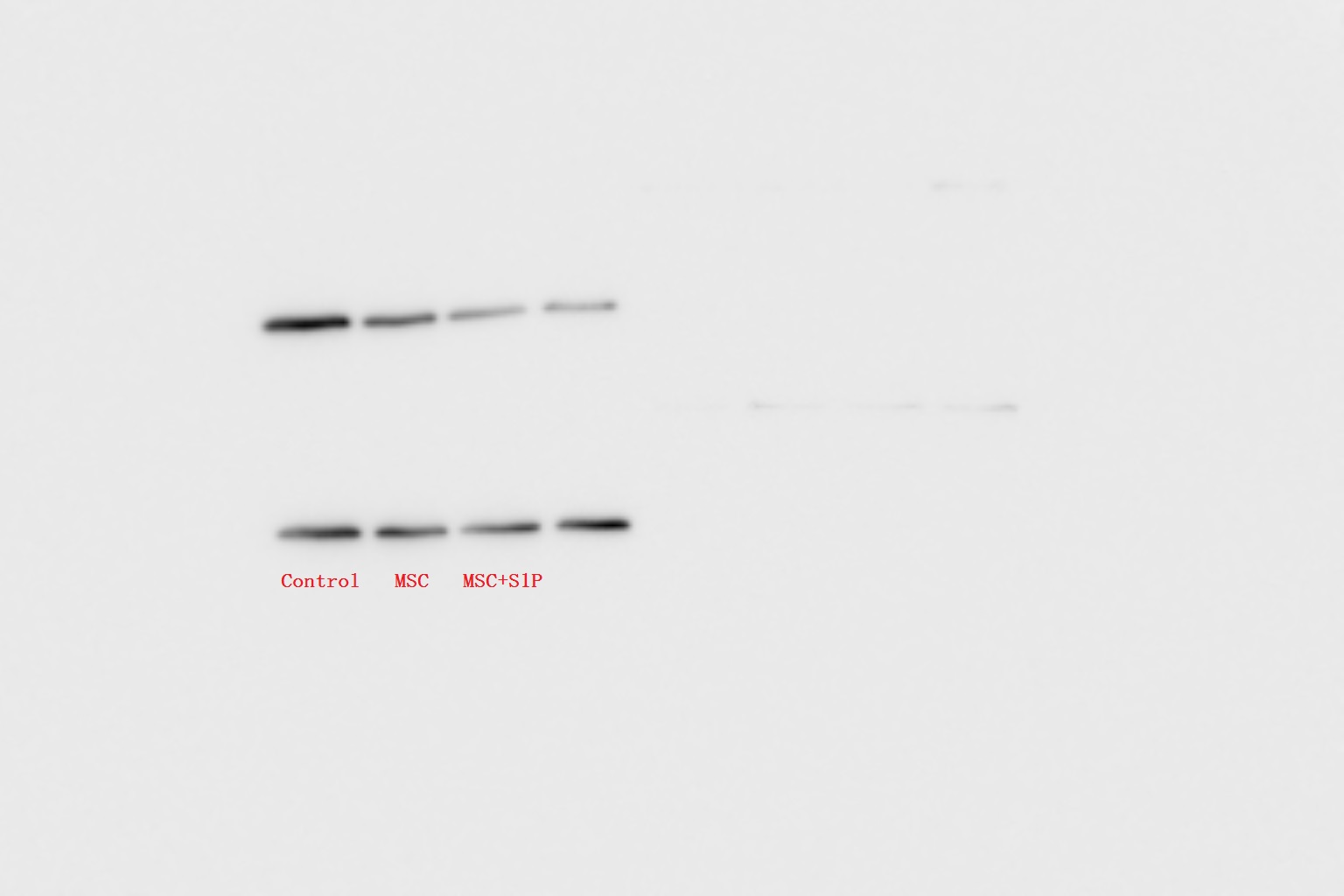

Supplement: Supplemental Information 6 [file peerj-04-2712-s006.zip › Supplement-S6/Fig6-actin.jpg]

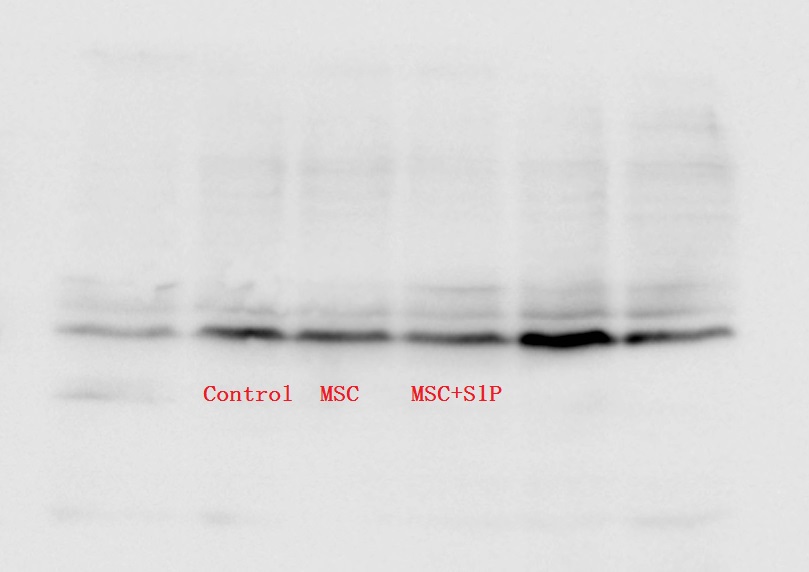

Supplement: Supplemental Information 6 [file peerj-04-2712-s006.zip › Supplement-S6/Fig6-S1PR1.jpg]

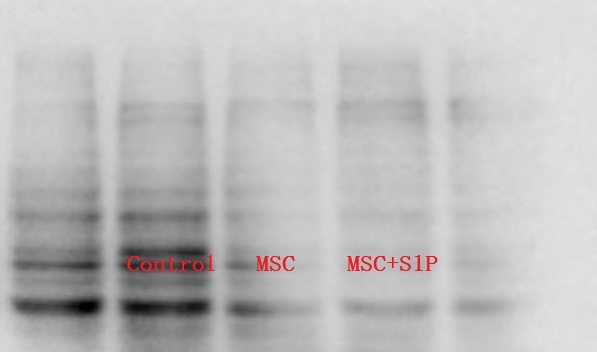

Supplement: Supplemental Information 6 [file peerj-04-2712-s006.zip › Supplement-S6/Fig6-S1PR2.jpg]

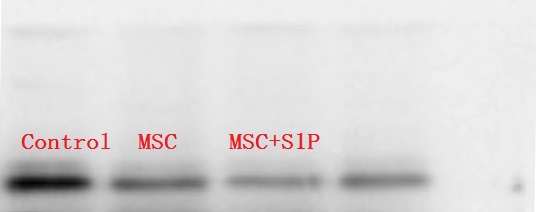

Supplement: Supplemental Information 6 [file peerj-04-2712-s006.zip › Supplement-S6/Fig6-S1PR3.jpg]
